# Paradoxical perception of humanistic care in the intensive care unit: A qualitative study

**DOI:** 10.1002/nop2.1399

**Published:** 2022-09-30

**Authors:** Fateme Hasandoost, Eesa Mohammadi, Mojgan Khademi, Mahyar Seddighi

**Affiliations:** ^1^ Faculty of Medical Sciences Nursing Department Tarbiat Modares University Tehran Iran; ^2^ Social Determinants of Health Research Center Lorestan University of Medical Sciences Khoram‐Abad Iran; ^3^ Anesthesiology and critical care Qazvin University of Medical Sciences Qazvin Iran

**Keywords:** humanistic care, intensive care units, nursing, qualitative research

## Abstract

**Aim:**

The aim of this study was to explore the perceptions of nurses, patients and attendants of humanistic care in the intensive care unit.

**Design:**

The present study was a qualitative conventional content analysis conducted in the intensive care unit in a hospital in Iran, in 2019.

**Methods:**

Data were collected using semi‐structured interviews and field notes through purposive sampling with 17 nurses who worked in an adult ICU in a teaching hospital, 4 attendants and 4 alert patients, and then analysed using the Elo‐Kyngäs method in 2008.

**Results:**

Analysis of the data led to the extraction of Four main themes as follows: (1) Insufficient understanding of nurses and patients' families of each other's roles, needs and expectations; (2) The use of personal and situational reasoning rather than ethical principles; (3) Caring stagnation; and (4) Satisfaction with care.

## 1 INTRODUCTION

Human caring in nursing is not considered solely as a feeling, approach, or even what one kindly desires to possess. From the moral point of view, caring in nursing is an ideal whose aim is to protect, enhance and preserve decency. It is, therefore, an ethical and philosophical enterprise defining nurses and individuals over time and in various conditions (Watson, 2012).

The utilization of humanism in clinical medicine leads to the potential evolution of patients' care, workplace culture and clinical education (des Ordons et al., 2018). The analysis of theoretical and research evidence indicates an urgent need for refocusing attention on human care and strengthening the humanism in healthcare systems, especially in nursing, as the largest health profession which plays a leading role in healthcare systems. Therefore, preserving and promoting human values in nursing can be a step towards strengthening these values and humanizing the whole healthcare system in a country (Khademi, 2013).

In contrast, dehumanization implies depersonalization, in which the illness treatment is viewed as the sole target of therapeutic care. A few out of many causes of dehumanization are the “dictatorship of technology,” which ends in considering patients as “things,” super‐specialization, the application of economic value‐based criteria to healthcare administration, and the refutation of the idea that the endurance and death of patients can affect relatives and experts equally (De la Fuente‐Martos et al., 2018).

Dehumanization versus humanistic care can be realized best in intensive care units (ICU). Patients admitted to the ICU have critical conditions and vulnerabilities. The ICUs with high‐tech equipment can cause job burnout for nurses and as a result, the nurses working in highly technological environments might abandon the humanistic aspects of their patients' care (Wilkin & Slevin, 2004). Recent scientific advances, coupled with increased awareness of cultural and social diversity, have led to a range of available treatment options; however, such developments sometimes lead to an adverse imbalance between science, technology and humanism in the clinical practice and has made humanistic excellence a major challenge in nursing (Mojgan Khademi et al., 2012; Sueiras et al., 2017).

### 1.1 Background

Due to the technical nature of the intensive care unit, the concept of patient‐centred nursing cannot be applied in its true sense in the field of intensive care. Technology strongly affects the patients' physiological status and violates patient‐centred caring (Jakimowicz & Perry, 2015; McGrath, 2008). Hence, recognizing two abilities technical and emotional should be adjusted and fused in critical care. The scholarly discussion has emerged over the strain among caring and technology (Locsin, 2005; Locsin & Ito, 2018; Luiz et al., 2017; Olausson et al., 2014).

What makes humanistic excellence a major challenge in nursing is the inconsistency of some aspects of modern healthcare and human guidelines (Khademi et al., 2012).

Evidence shows that most patients experience non‐human behaviours such as lack of compassion, concern and affection while they need to be cared for (Wiman & Wikblad, 2004). It indicates that the well‐being framework is itself a source of suffering (Arman et al., 2004).

In this regard, studies have been published about critical care nurses' experiences of providing care for adults; Crilly et al. in qualitative evidence synthesis found three analytical themes: sometimes machines get all the attention, with experience the patient becomes the focus and technology cannot save everybody (Crilly et al., 2019). Extensive research has shown that a number of qualitative studies provide nurses’ efforts to find balance when caring in a fully technological environment (Almerud et al., 2008; Martins et al., 2015; McGrath, 2008; Olausson et al., 2014; Stayt et al., 2015; Wilkin & Slevin, 2004).

In line with differences in care perspectives, Bagherian et al. showed a positive relationship between care characteristics and the effect of technology in the nurses. Care feature scores were higher in single female nurses. Although scores of care characteristics decreased with age and work experience, care commitment was higher in older, more experienced nurses. In addition, female nurses had a better view of the effect of technology on care. In contrast, young and experienced nurses had a negative view of the effects of technology on nursing care (Bagherian et al., 2017).

According to the findings of the reviewed studies, the effect of cultural and organizational conditions on the experiences and demonstration of humanistic care and the described situation of ICUs in the study environment, it is important to understand the importance of humanistic care in every field by taking into account the cultural and structural differences to enable and contribute to better humanistic care. To this purpose, this study attempts to explain perceptions of nurses and patient's families of humanistic care in intensive care units.

### 1.2 Design

A conventional qualitative content analysis approach was used in the present study. As naturalistic paradigms and qualitative methods take into account the dynamic and multiple nature of the reality under investigation and consider multiple constructs of a phenomenon as possible, they are believed to be useful to study the lesser‐known domains (Priest, 2006; Speziale et al., 2011).

## 2 METHOD

### 2.1 Setting and Participants

The present study was performed in the intensive care unit (ICU) of Bou Ali Sina educational and Medical Center is affiliated with a 70‐year‐old University from November 2018 to May 2019 in Qazvin city in Iran. This referral centre provides specialized and sub‐specialized services to members of the community and includes general and specialized departments. In ICU ward, patients with critical conditions related to internal diseases, nerves and poisoning are admitted and hospitalized. This ward has 11 active beds, all of which are considered as a separate unit. Also, one of the beds is located in an isolated room.

The human resources of the ward include a head nurse, Staff, who are present in the morning and non‐holiday shifts in the ward. This department has 4 service personnel in each shift, two of whom are women and two of whom are men, and they are responsible for transporting the patient, cleaning the patient daily and changing positions under the supervision of a nurse. Patients in this ward are visited daily by an anaesthesiologist who resides in the ward in each shift and is visited by an internal medicine specialist. Based on the presence of cardiac, infection or poisoning, counselling and visits by other physicians are performed. The task division in this department is case method, and in each shift, on average, each nurse is responsible for caring for 2 patients. Nurses are classified in A, B, C and D based on work experience. In this way, nurse A, who is higher than other staff in terms of work experience, is responsible for caring for 2 patients, and nurses B, C and D are responsible for caring for 3 patients. In this ward, visiting and attending the patients' families is prohibited, and 2 days a week, on Sundays and Thursdays, one of the main companions or relatives can visit the patient closely for 5–10 min, observing the principles of hygiene. At the time of the visit, the main companions are allowed to enter the ward, one to three with a short time interval, and the other companions can see the patient through the windows of the ward, which faces the hospital yard and the curtains are drawn at this time. This ward does not have a waiting room for patients' families and a glass room for visiting patients.

Data were collected using semi‐structured face‐to‐face interviews through purposive sampling with 17 nurses who worked in ICU and 4 attendants who were primary caregivers and had direct contact with nurses and physicians and 4 alert patients (Glasgow Coma Scale (GCS = 13–15)) and had hospital length of stay more than 48 h. The key characteristics of the participants are displayed in Table 1. Each interview last approximately 25–120 min. Field note observations were also recorded.

**TABLE 1:** Characteristics of the participants

Number of participants: 25 Interview duration: 25–120 min
Family: 4 Age: 41.25(21–53) Gender: 1 female and 3 males Education: Illiterate: 1, High school diploma: 2, Student: 1 Job: House Keeper: 1, Worker: 2 Family relationship Spouse: 1 Child: 2 Parents: 1
Patient: 4 Age: 35.7(17–53) Gender: 2 females and 2 males Education: High School: 1, High school diploma: 2, Bachelor: 1 Hospitalization: Yes: 3, No: 1 Condition: Poor: 1, Moderate: 1, Good: 2 Kind of disease Respiratory and cardiac problems: 1 Metabolic disorders (diabetes, poisoning): 2 Nervous problems (Guillain‐Barré syndrome): 1
Nurse: 17 Age: 33.68 (21–45) Clinical experience: 10.31(1–21) Education: Bachelor: 14; Master: 3 Position: Nurse: 13, Head Nurse: 1, Staff: 1, Supervisor: 1, Faculty member:1

### 2.2 Data collection

Semi‐structured interviews were conducted with nurses and patients and families to collect the data. All the interviews started asking an open‐ended and general question from the participants. The questions of the interview guide were general (Box 1). Interviews continued until data saturation was reached and no new codes could be identified (from participant 25). Field note observations were also recorded in the intensive care unit to complete and deepen the interview data. They are intended as objective evidence that gives meaning and aid in the understanding of the humanistic care phenomenon. Field notes allowed us to access the perceptions of the patients and record what they observed unobtrusively.

BOX 1 **Interview guide**

**Table:** 

Nurse	Patient/Family
Please, explain how you take care of your patient in this unite? Based on your daily experience, how do you try to provide is care humanely? In what situations did you feel these humanistic behaviours were lessened? For probing; Can you give an example? Explain more about this? What does this mean and what do you mean?	Please, explain the behaviour of ward nurses in caring for you/your patient? Please explain how you felt about these nurses' behaviours? In what circumstances these humanistic behaviours improved? In what circumstances did you feel these human behaviours are decreased? For probing; Can you give an example? Explain more about this? What does this mean and what do you mean?

### 2.3 Analysis

The data obtained from the interviews were analysed after they were recorded and transcribed word by word according to the Elo‐Kyngäs method (Elo & Kyngas, 2008). This method includes three steps: 1. Preparation, 2. Organization and 3. Abstraction. During the preparation stage, the whole interview, which could be a good context for semantic units, was selected as the most appropriate unit of analysis. Each interview was read several times so that the researcher could go deeply into the participants' perceptions. At the organization stage, open coding was performed by re‐reading the interviews, taking notes and writing labels on the margins. The labels were then recorded on the code sheet. Grouping began after several interviews were read. Additional labels and categories evolved by repeating the abovementioned stages for each new interview. After comparing and merging categories belonging to a group, the number of initial categories was reduced. The sub‐categories with similar themes were grouped as one category. Each category was then named by words representing its content characteristics. The abstraction process continued until four main themes were extracted (Table 2).

**TABLE 2:** An example of the theme extraction process

Quotation/Field notes	Initial codes	categories	Theme
Bed 9 as a bradycardia patient and attracted my attention. I informed the nurse and I said you were doing nothing for Bed 9. The nurse said he was expired. His family had a wedding today and we injected him atropine to get him alive until the end of wedding, They demanded the nursing office not to ruin the wedding if possible…(Field note 28)	The nurse's attempt to save a patient at the request of the family and the nursing office for a longer period of time	Facing moral contradictions in saving life	The use of personal and situational reasoning rather than ethical principles
Taking care of patients with a low level of consciousness is useless and only takes our time and energy. Our colleagues say if he is sent to the ward, he will be soon expired (Nurse Ms. N)	Helping the expedition of deaths of irreversible patients and the final stage		
An internist came to the ward today and the head nurse said: Doctor, you should send the bed 10 patients, who are being in a bad condition, to the general ward where they should decide what to do. They insisted, but the physician did not accept to send the patient to the ward. The patient had severe rectal bleeding…The doctor said how I could send the patient…The head nurse said: He would waste a lot of money and energy. They are too skinny, and we have nothing to give them, so they become thin and catch sore. (Field note 10)	The physician's refusal to comply with the nurse's request to expedite the death of an irreversible patient		
We didn't care about old patients or those who had lower levels of consciousness. I spend my time and energy on those who are more likely to be recovered. (Nurse, Ms.Kh)	More care for patients with a high likelihood of recovery and a higher level of consciousness	Caring preference in young and conscious patients	
If a patient is more conscious, younger, or entered unwanted to this ward, like the suicide patients, and it is the main cause of their death, when they are put under the ventilated machine, they say please save us, we want to live, I am regretful, so the caring is done further. These cases give us the motivation to provide care to treat them (Nurse Ms. Gh)	More human attention to regretful and young suicide patients		

### 2.4 Rigour

Long‐term engagement of the researchers for a year in the field and spending enough time communicating with participants during data collection helped the researchers to build trust and understanding in participants and enabled deep data collection. According to Lincoln and Guba's criteria (Ryan et al., 2007), maximum variance sampling is based on features such as age, work experience and the position was used to confirm the transferability of the findings. To ensure that the analysis accurately reflected the participants’ experiences, the codes extracted from each interview were controlled and reviewed by the participants during data collection and analysis, and necessary changes were made in data interpretations based on participants’ suggestions. To provide dependability and confirmability of data, two nurses who were expert in conducting research and had working experience in the intensive care unit reviewed parts of the raw data including interviews and analytical products, namely initial codes and categories.

### 2.5 Ethics

Code of ethics (IR.TMU.REC.1397.182.) was obtained from Tarbiat Modares University, and the research environment licence was received. The importance, purpose and method of research were explained. The interviews were recorded. Participants' informed consent obtained was written. Confidentiality and anonymity at all stages were met and the time and place of interviews were mutually decided. The participants were free to participate in or to withdraw from attendance. The researcher's characteristics and the way of access to results were explained to the participants.

## 3 RESULTS

Analysis of the data yielded 367 initial codes, 16 categories and 4 main themes including “insufficient understanding of nurses and attendants of each other's roles, needs and expectations,” *“*the use of personal and situational reasoning rather than ethical principles,” *“*caring stagnation” and “satisfaction with care.” The extracted themes indicated that nurses and attendants did not have sufficient understanding of each other's roles, needs and expectations due to the existing conditions in the ward and that the nurses neglected the professional ethics in caring and employed personal reasoning. Caring stagnation was found in the observations of caring behaviours and their interpretations. However, under the critical conditions of the patients and the ward, the nurses sought to provide humanistic care and satisfied the patients and themselves and they felt satisfied when being in and out of the ward.

### 3.1 Insufficient understanding of nurses and attendants of each other's roles, needs and expectations

The findings revealed that the attendants insisted on tracking the status and treatment of their patients and were willing to be present at their patients' bedside and cooperate in the care procedure. However, the attendants were neglected by the personnel and their presence was considered unnecessary and obtrusive to patients' care. The nurses were unable or unwilling to communicate and inform them (attendants). It was indicated that nurses did not understand the needs and desires of attendants, and the attendants did not understand the nurses' working conditions, measures, constraints and needs. This theme has five attributes including “families' insistence on the follow‐up of situation and treatment.” “Ignoring families.” “Unnecessary and disturbing presence of families.” “lack of skill and willingness to interact and guide” and “voluntary participation in care.”

### 3.2 Families' insistence on the follow‐up of situation and treatment

Limiting the visiting hours to 2 days a week for a quarter of an hour in the intensive care unit was a major challenge for the families. The families were allowed to visit the patient, but, nurses were opposed to the family to meet the patient.

“The attendant was looking from the glass door. I asked ‘What do you want?’ he said ‘Nothing I just want to see my son.’ …I said, ‘It's not the visiting time’. He said ‘I can see through the window if possible’. He could see his son through the window with the coordination of ward nurses and happiness could be seen on his face” (male 72‐year‐old family 2).

### 3.3 Ignoring families

During the patients' care, their attendants are often forgotten. It was found that the attendants' needs are forgotten and they are left without any support, education and caring programmes.

“A patient's attendant came to see her patient and started crying. The service guard shouted and told her not to cry there. She took her cry with herself and went to the nursing station to ask a question. The nurse did not look at her and said ‘I do not have time’ …and then the attendant left the ward”(Field note 1).

### 3.4 Unnecessary and disturbing presence of families

The participants' experiences revealed many events, beliefs and attitudes affecting the nurses' humanistic performance, such as the belief in the uselessness and harmful presence of family members at the patient bedside, the consideration of family members as those with low health literacy, requiring them to spend extra energy to justify the attendants, and disturbance in care processes due to the presence of families.

“Unfortunately, the level of our people's culture is low, and they lack health literacy and if we want to tell them what is going on, you have to spend your time and energy” (Nurse Ms. A).

This complaint about the lack of understanding is mutual. In other words, the attendants complain about being ignored, and the nurses complain that the attendants do not understand their professional roles and responsibilities, and thus, they consider their presence disruptive to their professional duties.

### 3.5 Lack of skills and willingness to interact and guide

Communicating with and informing the attendants about the patients' conditions in the intensive care unit are a highly important issue that is often overlooked. Some nurses are unable to provide appropriate care or are reluctant to perform their professional duties owing to their inability and lack of interactive and educational qualifications. This can cause major problems such as conflicts, complaints, fears and doubts about decision‐making for families and attendants.

“The ward nurse asked for the consent of tracheotomy from the attendant of bed 3. The attendant was confused and did not know what the tracheotomy was. The more the nurse tried to explain, the little she understood. There was no one in the ward to have the tracheotomy, therefore, the attendant was scared and said that “I must consult with my family and I could not decide alone” (Field note 2).

### 3.6 Voluntary participation in care

Contrary to the previous characteristic, many attendants frequently visit the care unit and query the staff in order to help with the patients' care. The field observations indicated that some attendants asked the nurses if they could cooperate in caring. Some nurses also said that attendants had a strong desire to improve their patient status; therefore, they were more alert and could attend the ward whenever needed.

“A young girl was suffering from hypoxia following a cardiac arrest. The patient's husband, who was also very young, was trying to use the olive oil to make her skin greasy to prevent scarring. He frequently asked questions from nurses what you need to prepare for the patient and was often present in the hospital” (Field note 3).

### 3.7 The use of personal and situational reasoning rather than ethical principles

Some findings indicated that ethical decision‐making is challenging in complex conditions of the patients in the ICU. Factors such as complexity of the situation, tendency to save energy and time in difficult working conditions, nurses' emotions and feelings, demands of patients' families and the supervisors' orders complicate the process of reasoning and ethical decision‐making by nurses. Replacing ethics with personal and situational reasoning was experienced in two ways explained in (Table 2).

### 3.8 Caring stagnation

This theme indicates that the variety and intensity of work stress in the intensive care unit and neglect of nurses' roles and rights lead to insufficient efforts by the nurses to meet all the needs of patients in the intensive care unit, nurses' fatigue and mistrust. This theme is included “insufficient nurse care to meet all patients' needs in the intensive care unit,” “patients' mistrust in the nurse's sayings,” “neglect of the nurses’ roles and rights,” “diversity and intensity of work stresses in the intensive care unit,” and “focus on the unconscious patient's body. Many nurses try to do many caring affairs carelessly and routinely in response to not being seen in the workplace. When nurses realize that there is no difference between them and other nurses who deliberately or undeliberately do clinical affairs carelessly, they change their attitudes to their job. This issue sometimes leads to care stagnation.

### 3.9 Insufficient nurse care to meet all patients' needs in the intensive care unit

There is an inadequate attempt of nurses to meet all the needs of patients in the intensive care unit such as nutrition and skin care so that insufficient supervision was exercised after delegating authority to the unprofessional staff.

A patient was saddened by seeing the death of others.

“Well, everybody here is dying every day, and they cover the patients and take them; and I think they are waiting in line. It is ordinary for very ordinary nurses, but it is difficult for me, and it affects my spirit. I am not used to it as them” (Patient, a 43‐year‐old man with Guillain‐Barré syndrome).

### 3.10 Patients' mistrust in the nurse's sayings

Some patients, who are hospitalized for a long time in the ward, lose their trust in nurses and insist and repeat their demands.

“It was the last hour of my shift when I visited bed 9 for saying goodbye. The patient was under tracheotomy and was conscious. She said, ‘I want sweet tea’. I prepared tea with the help of a service worker and a nurse. She said, ‘Give it to me, but it was too hot’. The nurse told her to let it to become cold, and then we take it. She said ‘No, just now’. I told her that it was hot and showed it. The nurse saw that she was upset and poured some tea with a syringe into her mouth and gavaged the remaining” (Fieldnote 7).

### 3.11 Neglect of the nurses' roles and rights

Cases such as stressful and difficult working conditions in the intensive care unit, lack of staff and overwork cause nurses to feel that managers neglect their rights.

“The shifts are better now, but fewer working hours are effective. Nurses should have a regular program of entertainment. The shortcomings should be overcome. When for example, there is a lack of drugs, no devices and doctors take our time for calling and finding a physician. If you come home late at night, I cause much stress; you have left the kids, and your life and had these tensions, or the number of beds for nurses should be decreased, and the routine of ICU should be adhered” (Nurse Ms. D.).

### 3.12 Diversity and intensity of work stresses in the intensive care unit

Some nurses pointed out the impact of environmental stressors on patients' care.

“For example, my personal experience is that when stress comes to life, my whole day collapses, everything is messed up, my patient work and everything gets messed up, but on days with more energy for patients, I'll feel that the patient's clinical situation would become better” (Nurse Ms. M).

### 3.13 Focus on the unconscious patient's body

Most nurses focus on the physical care of patients and forget communication with non‐conscious patients.

“We had a mental patient, we were asked to talk to her quietly, have good behavior, and explain what we were going to do for her, but we don't give an explanation for the unconscious patient, because we think it is unnecessary” (Nurse Ms. N.).

### 3.14 Satisfaction with care

This theme is characterized by “gratitude and spiritual excellence from receiving internal and external feedback,” “answer to the call of conscience,” “patient‐nurse mutual consideration and appreciation” “sensitivity to patients' needs and vulnerabilities.” Although nurses work with high pressure and are overworked in the intensive care unit, they become satisfied with patients whose care is successful and in case of patients' improvement as well as positive reactions such as patient and family appreciation.

### 3.15 Gratitude and spiritual excellence from receiving internal and external feedback

Nurses believe that patient care is accompanied by spiritual rewards and benefits for themselves and their families.

“When you take care of a patient who is not conscious, you provide care and know God knows, but if a patient is conscious and prays for you, you become so happy. For example, the patient says God bless you and protect your children, and patients become happy with you…, all these things affect your life and are useful for your children and family” (Staff Ms.N.).

### 3.16 Answer to the call of conscience

Nurses noted a feeling of torment after failing to perform clinical measures for patients.

“In fact, I had no special thought before, even when I was a student, I studied indifferently, but I thought what happened if one of my beloved ones became ill, so I found that the Karma exists. It is easy to abdicate the responsibilities, but the sense of responsibility does not allow doing everything” (Nurse Ms. Kha).

### 3.17 Patient–nurse mutual consideration and appreciation

Apology to patients after performing aggressive procedures and the patients' appreciation of care provided by nurses led to the creation of this category.

“Since I was long working in the intensive unit, for example, when a patient was in sleep mode, I put my hand on her shoulder and say dear mother I am putting your suction; dear mother I am doing the venipuncture. Additionally, when I search for an arterial blood vessel and cannot find it, I'll apologize for being annoyed…” (Nurse Ms. Agh.).

### 3.18 Sensitivity to patients' needs and vulnerabilities

Nurses complain about the inability to resolve the patients' problems.

“There was a young woman who had breast cancer. She had the worst possible expire. She was in my mind due to her chemotherapy and septicemia. She was conscious until the last moment, and the tracheotomy was totally performed for her. We had a lot of sympathy for her. She had reflux and could eat nothing. She was thirsty and we could give her nothing, and we saw his gradual death” (Nurse Ms.N.).

## 4 DISCUSSION

The findings indicated that the nurses and participants (patients' families) had insufficient understanding of each other's roles, needs and expectations. Also nurses use of personal and situational reasoning rather than ethical principles in care. While caring stagnation is perceived, nurses and patients' families experience a sense of satisfaction with humanistic care. Researchers in other studies considered the perceptions of care providers and recipients mostly in cases where patients had sufficient consciousness, but it was less performed in the intensive care unit and particularly with patients with insufficient consciousness.

In line with the inadequate understanding of nurses and attendants roles, needs and expectations, Martos et al. in Spain indicated that professional performance in medicine and activity in intensive care improved communication not only among caregivers in that ward but also with caregivers in other wards of the hospital and patients and their families. Furthermore, patients and their families promoted caring motivation by caring tools and helped to promote humanistic care in the intensive care unit (De la Fuente‐Martos et al., 2018). The results contradicted the findings of the present study as a category “Lack of skills and willingness to interact and guide” between nurses and families in the intensive care unit.

Moreover, Wong et al. conducted a grounded theory study indicating that families of patients in the ICUs primarily sought information and awareness in their interactions with the nurses. Employees sought supportive ways to interact with families owing to interpersonal communication and skills (Wong et al., 2015). Although the two studies by Martos et al. and Wong et al. achieved notable success in understanding and responding to the needs of families in the ICUs, different changes can be made in any environment depending on organizational and cultural conditions (De la Fuente‐Martos et al., 2018; Wong et al., 2015). It is necessary to use the experience of others, including these studies, and perform appropriate interventions to resolve the problem according to organizational and cultural conditions.

One of the findings of the present study was “Unnecessary and disturbing presence of families” and “Ignoring families” relating to the following theme, “insufficient understanding of nurses and patients' families of each other's roles, needs and expectations.” Unlike the present study, other studies emphasized that health is based on a patient‐centred and family‐centred system (Davidson et al., 2017; Khaleghparast et al., 2016). Spreen et al. explained the principles and standards of visiting in the intensive care unit, stating that the “open visit” policy was an urgent need for patients and families in the intensive care unit. Clinical guidelines in many countries recommend open care policy in the intensive care unit in line with family‐centred care (Hasandoost et al., 2018; Spreen & Schuurmans, 2011). Other studies also indicated that families needed participation, and their presence was not only a nuisance but also useful and effective (Abbasi et al., 2009; Salmani et al., 2017).

Despite these standards and valid findings and evidence, in Iran, there are still restrictions on open visiting that need to be standardized by training or removing organizational barriers.

As related to the theme, “The use of personal and situational reasoning rather than ethical principles”, the findings indicated that nurses were sometimes influenced by emotional or cross‐sectional demands of families, and in ethical conflicts, they acted personally with outcome‐centred and emotional reasoning.

It is always emphasized that in the adherence to professional ethics, patients have rights in the healthcare system that must be respected and protected, and this cannot depend on providers' personal preference and desire (Sanjari et al., 2011).

Consistent with the findings of the present study, “Facing moral contradictions in saving life” in one study by Falcó‐Pegueroles et al. found ethical conflict is an intrinsic problem, but it is strongly influenced by specific variables and environmental conditions. Nurses' participation in decision‐making when patients have critical conditions appears to be a protective factor against ethical conflicts (Falcó‐Pegueroles et al., 2016). It is important to note why nurses sometimes tend to like the earlier death of patients or, in some cases, make the patients alive with medical interventions due to the family requests; why they are more inclined to serve the youth, and, on the contrary, the elders are less considered. These two concepts seem to be particularly internalized in the intensive care. Although it creates apparent logical reasoning for nurses, it causes ethical challenges and internal conflicts, and thus makes nursing less humanistic and without the purpose of being good and peaceful death. Chamberlin found that useless or potentially inappropriate care was associated with neglecting patients, relatives and colleagues, and burnout‐related behaviour (Chamberlin et al., 2019). Since, in some cases, nurses are confronted with irrational demands of the patient's family, understanding the concept of humanistic care (Wilkin & Slevin, 2004) helps them make appropriate clinical decisions.

Another theme found in this study was “caring preference in young and conscious patients.” We can address one study by Khademi et al.(Khademi et al., 2017) indicating the severity of disease threat, vulnerability and weakness of supportive resources. Such conditions (age groups, such as children and the elders, and the absence of family members at patients' beside) mean the need for careful and long‐term care. Unlike the present study, this may vary based on the nurses' personality traits that are nurse‐related factors in human care delivery, namely care definition, differentiation, absent nurses, analysis orientation, routine orientation, restriction and invisibility in the hourglass model (Beltran Salazar, 2016).

Consistent with the findings of the present study, “care preference in young and conscious patients,” Laerkner et al. (2015) indicated that despite the complexity of care, nurses preferred taking care of conscious patients.

Consistent with our findings, insufficient care by nurses to meet the needs of all patients in the ICUs is a characteristic of caring stagnation. Khademi et al. indicated that claims and unsuccessful self‐compensation for rights occur overtime after the right violation (Mojgan Khademi et al., 2012). The violation has three main dimensions, namely “caring stagnation”, “mechanical care” and “disrespect”; and patients' rights—as social rights—determine the quality and access to health care (Khademi et al., 2019). The rights violated in hospital settings can lead to patient/family rights violations and care stagnation. Salazar states that many factors can lead to impersonal care in organizational or personal contexts based on the hourglass model. Humanistic care can be changed to impersonal care or vice versa depending on the orientation of nurses in caring practice and some elements in the organizational context (Beltran Salazar, 2016).

Another finding of our study was “satisfaction with care,” which was aligned with the findings of Khademi et al. In some cases, nurses are involved with the process of unsparing response to situations that are influenced by the synergy of education/learning and situation. In addition to satisfaction, especially receiving care benefits and rewards, it also means achieving an aspect of spiritual rights and satisfaction (Khademi et al., 2017).

In line with the findings of our study about the “patient‐nurse mutual respect and appreciation,” an ethnographical study by Laerkner et al. found three main themes including “a sense of ability,” familiarity with unknown situations” and “being conscious of their surroundings” (Laerkner et al., 2017). The patients were able to interact and gain a sense of ability since the early days of the disease. They appreciated the compassionate and caring nurses who cared for them and involved them in affairs. The patients were aware of the activities around them, felt unable while being neglected by staff and were affected by the sufferings of other patients. They were interconnected as they woke up to their inability and lack of power alongside conflicting factors such as physical weakness, technology, location and communication aspects.

## 5 LIMITATION

There is an opportunity of bias in data collection, analysis and announcement of results. The researcher with Bracketing their background and knowledge and presenting open and general questions during the interview that participants have the opportunity to express their perceived experience and frequent observations and attendance in patient care so that they feel the researcher is their partner. It prevented the occurrence of artificial and inductive behaviours and also tried to avoid bias in the data by using the techniques expressed according to the Lincoln criterion. At the same time, the subconscious may be biased, which is negligible in qualitative study.

## 6 IMPLICATIONS AND RECOMMENDATIONS FOR PRACTICE

Inadequate understanding of the patient / family and nurses of each other's needs, expectations and tasks informs ward and hospital managers and health policy makers that they need to take action and develop policies so that, first, the family better understands the conditions of intensive care and nursing careers and sets their own expectations. Also inform nurses and ward managers on how to empathize and inform the patient and the patient's anxious and worried families. Caring stagnation as a warning in clinical practice leads nurses to burnout and seeks to demand and unsuccessful compensation of their rights. By defining tasks and expectations and increasing motivation, it can be greatly helped to prevent it. Also, the use of personal and situational reasoning rather than ethical principles by not paying attention to the observance of codes of professional ethics and the need to re‐emphasize it by nurses with participatory decisions are the most important issues and barriers in humanistic care in the intensive care unit, in order to strengthen and make satisfaction with care in nurses through measures and interventions, and to improve the quality of care and make care more humane.

## 7 CONCLUSION

The findings of this study in the form of four themes, although expressing the perception and experienced meaning of nurses along with patients and their families of humanistic care, but at first glance are paradoxical and inconsistent in nature, but their analysis and explanation in the discussion section show justification and is their logical harmony. So that, the concept of care stagnation indicated that the humanistic care in the intensive care unit did not emerge and was not provided with its true essence and meaning. An underlying cause of this issue was the nurses and families' inadequate understanding of their mutual roles, needs, expectations and even limitations of each other, since it allowed nurses to consider personal and situational reasoning based on the limitations rather than on ethics and patients and attendants' needs and preferences, resulting in feeling and understanding the care stagnation. Despite this result, nurses feel an unexpected feeling contrary to the nature of care, which is satisfaction, due to experience and observation of patients' health improvement. Although this paradoxical feeling is the outcome and implication of the care process, it may be the result of changes in the conditions of ICU patients.

## AUTHOR CONTRIBUTIONS

Hasandoost: Interaction with participants, interviews performance, transcriptions, the initial analysis and writing the initial draft with contributions from all authors during the editing procedure. Mohammadi & Khademi: Conceptualization and design of the project, supervision and guidance of data collection and interview and their analysis, modification and completion of the draft of the article. Seddighi: Assist in collecting and analyzing data and writing articles.

## FUNDING INFORMATION

The author(s) received no financial support for the research, authorship and/or publication of this article.

## CONFLICT OF INTEREST

The author(s) declared no potential conflicts of interest concerning the research, authorship and/or publication of this article.

## Data Availability

The paradoxical understanding of humanistic care leads to the dissatisfaction of the beneficiaries.
